# 248. Outcomes of Patients with Prosthetic Septic Arthritis with Debridement and Implant Retention

**DOI:** 10.1093/ofid/ofab466.450

**Published:** 2021-12-04

**Authors:** Rattanaporn Mahatanan, Antonia Altomare

**Affiliations:** 1 Dartmouth Hitchcock Medical Center, Lebanon, New Hampshire; 2 Dartmouth-Hitchcock Medical Center, Lebanon, New Hampshire

## Abstract

**Background:**

IDSA has published guidelines for the diagnosis and management of prosthetic joint infection (PJI). However, we have observed significant variability in the interpretation and application of these guidelines with respect to the management of those with PJI following debridement and implant retention (DAIR). It is not clear if variations in antimicrobial management are affecting clinical outcomes.

**Methods:**

We performed a retrospective review at an academic hospital in rural New Hampshire. We included all adult patients from 1/1/2017 to 12/31/2018 with PJI of hip or knee who underwent DAIR. The demographic data, microbiology data, antibiotics treatment and duration were collected. The primary endpoint was overall re-infection rate within 2 years of surgery. Secondary endpoint was re-infection rate stratified by organism and antimicrobial type and duration.

**Results:**

A total of 26 patients were included in our study. 65% involved knee joint. 50% had late-onset infection ( >12 months). The top organisms were Streptococcus spp. (34%), CoNS (26 %) and MSSA (18 %). 15% were associated with bacteremia. Ceftriaxone was the most common antibiotic used (54 %). 38 % of patients received Rifampin PO along with IV antibiotics. All patients received PO antibiotic(s) after completing the course of IV therapy, and 7 patients were also on concomitant rifampin PO. The duration of PO antibiotic therapy was varied. 30% of patients received PO antibiotics for 6 months post IV treatment. Life-long suppression therapy were noted in 9 patients. Treatment failure within 2 years occurred in 8 patients (31%). Among those, 75% had Staphylococcal infection. All patients required hardware removal except one patient who required amputation. 2 patients developed recurrent PJI after completing 6 months and one year of PO suppression therapy, one patient had a recurrent infection while on life-long suppression. Staphylococcal infection was significantly associated with treatment failure.

**Conclusion:**

Treatment of PJI with DAIR is challenging. Despite long-term IV therapy followed by oral antibiotics, there was a high rate of treatment failure (31% in our study) particularly with Staphylococcal infection. There was no association of variation of treatments and outcomes in our small cohort.

**Disclosures:**

**All Authors**: No reported disclosures

